# Temperature discomfort impairs everyday cognition: a pilot study using smartwatch-based ecological momentary assessment

**DOI:** 10.1088/2515-7620/ae6239

**Published:** 2026-05-04

**Authors:** Kimberly L Meidenbauer, Catherine M Luna, Phillip John Stilson, Isabella M Santiago, Diane J Cook, Maureen Schmitter-Edgecombe

**Affiliations:** 1 Department of Psychology, Washington State University, Pullman, WA, United States of America; 2 School of Electrical Engineering and Computer Science, Washington State University, Pullman, WA, United States of America

**Keywords:** temperature, thermal comfort, cognition, *N*-back, EMA

## Abstract

High ambient temperatures are associated with a variety of negative outcomes, from exacerbated mental illness to aggression to increased dementia symptoms. One possible proximal mechanism influencing these impacts is heat-related changes in cognition. These effects of extreme heat on cognition have been widely investigated; however, the relationship between thermal discomfort at typically experienced temperatures and everyday cognition has received minimal attention. This pilot study evaluates the feasibility of a smartwatch-based ecological momentary assessment (EMA) design for effectively assessing this relationship. We examine whether thermal discomfort and distracting temperatures are sufficient to impair both objective (*N*-back task performance) and subjective (self-reported alertness) cognitive function. Results demonstrated that thermal discomfort led to worse performance on the *N*-back task. These effects were not affected by time of day but did show an interaction with acclimatization effects. The presence of distracting temperatures was also associated with lower scores on the *N*-back task. Taken together, the results of this pilot study demonstrate that deviations from comfortable temperature conditions can impair executive attention and cognitive control in daily life. Further, they highlight the utility of using combined EMA surveys and cognitive tasks to examine the effects of the physical environment on cognitive performance.

## 1. Introduction

As daily ambient temperatures increase due to climate change, there is an increasing call to understand the scope of heat’s detrimental effects on individuals’ mental and cognitive health (Taylor *et al* 2015, Lõhmus 2018). At a population level, hotter days are linked to increased drug overdoses (Roy *et al* 2022), emergency department visits associated with self-harm behaviors (Basu *et al* 2018, Nori-Sarma *et al* 2022), accidental injuries (Otte Im Kampe *et al* 2016), and suicide attempts (Yarza *et al* 2020). Aggression and violence increase with higher ambient temperatures (Miles-Novelo and Anderson 2019, Heilmann *et al* 2021, Choi *et al* 2024), and learning and academic achievement are also hindered during hotter weather (Park *et al* 2020). However, the proximal mechanisms driving these large-scale effects remain somewhat nebulous. While there are likely multiple causes, including disrupted sleep (Obradovich *et al* 2017, Mullins and White 2019) and worsened affect (Noelke *et al* 2016, Meidenbauer *et al* 2024), one particularly promising mechanism is heat-related impairments in cognitive functioning (Byrne *et al* 2020, Fischer *et al* 2024, Zhang *et al* 2024). As cognitive control and executive functioning are integral to healthy emotion regulation (Ochsner and Gross 2005, Hendricks and Buchanan 2016, Pruessner *et al* 2020) and impulse inhibition (Aron 2007, Peckham and Johnson 2018), impairments in these cognitive processes are likely to play a key role in the population-level effects.

Supporting this notion, a large body of experimental work demonstrates the acute effects of heat on higher cognition. These studies typically involve exposing healthy adults to relatively extreme exposures (43 °C–50 °C or 110° F–122° F) and measuring performance on a variety of cognitive outcomes, including tasks of decision-making, executive functioning, attention, memory, and processing speed (Racinais *et al* 2008, Taylor *et al* 2015, Shibasaki *et al* 2017, Gaoua *et al* 2018, van Maanen *et al* 2019). Notably, impairments of complex cognitive processes, particularly those that place high demands on executive attention, working memory, or impulse inhibition, appear to be more pronounced than those for simple, easy tasks (Taylor *et al* 2015). In cases where hyperthermia (fever) is induced, functional magnetic resonance imaging studies demonstrate alterations in the prefrontal cortex and other regions involved in the executive attention network (Shibasaki *et al* 2017). These alterations disrupt the functional connectivity of key neural networks, which coincide with worse behavioral performance on complex tasks (Liu *et al* 2013, Sun *et al* 2013).

However, most experimental studies use extreme temperatures, which are not representative of the daily conditions most people encounter. Indeed, the effects on cognition at more typically-experienced temperatures are less studied. A few experimental studies have tested high heat environments that do not lead to a change in core body temperature, but do increase feelings of discomfort. One study by Gaoua *et al* (2012) demonstrated that thermal discomfort in the absence of hyperthermia was sufficient to worsen performance on a complex cognitive task, and it was hypothesized that the discomfort may act as a ‘cognitive load’, distracting participants from completing their task (Gaoua *et al* 2012). Other studies have shown impairments in decision-making and executive functioning tasks at more modest temperature inductions (Decker *et al* 2014, Abbasi *et al* 2019, Donnan *et al* 2022, Lan *et al* 2022). However, the evidence at more modest heat is somewhat mixed, with some studies demonstrating null effects (Parker *et al* 2013, Schlader *et al* 2016, Stroom *et al* 2021). This suggests that the effects are influenced by heterogeneity in study design, sample characteristics, measurement type, and/or exposure dose and duration.

In addition to the mixed results, explicitly linking the immediate psychological consequences of heat stress to large-scale mental health outcomes faces another important challenge. That is, while these lab studies offer excellent experimental control, they are often lacking in ecological validity. It is likely that many of the effects on heat in the real world arise in large part from cumulative exposure or exposure over longer durations than is typically tested in the lab (1–2 h). Indeed, much of the population-level effects examine how changes in ambient temperatures at the level of days, weeks, or months impact well-being. For example, one recent study found that for each 1 °C increase in monthly temperature, older adults’ global cognitive function scores decreased by nearly 0.5 points (Hou and Xu 2023). Similarly, research linking heat exposure and emergency room visits for Alzheimer’s disease and related dementias found strong effects for cumulative exposures over the course of a 3 d period (Zhang *et al* 2023).

Additionally, relying on weather patterns to determine exposures is a challenge, as access to and use of indoor climate control can account for major differences in actual heat exposure. A recent study examining the effects of heat stress on city residents’ well-being showed that outdoor ‘objective’ exposure and indoor ‘subjective’ exposure (i.e. self-reported) were both individually related to participant-reported impulsivity. However, the outdoor and indoor estimates showed a negligible correlation to each other (*r* = 0.02; Meidenbauer *et al* 2025) and the indoor heat effects were more strongly related to this outcome, which may be due to the fact that North Americans spend roughly 87% of their time indoors (Klepeis *et al* 2001). It may also be that the subjective experience of thermal comfort matters more than the objective temperature. A recent study using ecological momentary assessment (EMA) demonstrated that participants’ thermal comfort predicted worsened emotional well-being, but the actual local weather conditions and participants’ perception of temperature as hot (distinct from comfort) had no effect (Meidenbauer *et al* 2024). However, it remains unclear whether the same is true for changes in cognition, as some studies using EMA have also shown associations between temperature and cognitive impairment but focused on subjective cognitive reports rather than objective tasks (Baniassadi *et al* 2024).

To address the limitations of subjective reports, EMA approaches are increasingly using mobile cognitive tasks to better assess fluctuations in cognition due to environmental and situational factors. While these tasks are better at capturing objective changes in cognitive processes than subjective reports, known limitations of many of these tasks include high sensitivity to practice effects (Haith and Krakauer 2018) or limited ability to detect within-subject variances (i.e. due to fluctuations in fatigue, alertness, mood; Cauchoix *et al* 2018). One exception which has been recently validated in EMA is the *N*-back task (Schmitter-Edgecombe *et al* 2024). The *N*-back task is well-suited for frequent use because it is short (45 s) and is sensitive to these fluctuations in psychological states and robust to confounding practice effects after the asymptotic baseline is reached (Schmitter-Edgecombe *et al* 2020, Dai *et al* 2023, Cormack *et al* 2024). Additionally, the *N*-back task requires a variety of cognitive control processes (Schmiedek *et al* 2009), including executive attention, working memory, and inhibition, and has been shown to recruit brain regions in the fronto-parietal control network (Jonides *et al* 1997, Herff *et al* 2014, León-Domínguez *et al* 2015, Meidenbauer *et al* 2021). Accordingly, as the *N*-back task relies on these higher-order cognitive processes, it is more likely to be sensitive to changes in temperature compared to simpler tasks (Taylor *et al* 2015), making it highly suited to the current study aims.

A key benefit of using an objective cognitive assessment in an EMA paradigm is that it enables the evaluation of cognition in context. That is, while the central aim of this work is to investigate the effects of thermal discomfort on cognition, there are undoubtedly other factors that independently impact cognitive functioning or may interact with thermal comfort to produce changes in cognition. For example, alertness and fatigue fluctuate over the course of the day, and the body’s circadian rhythm leads to changes in body temperature, which may impact comfort (Wang *et al* 2018). Acclimatization in the summertime, which reflects a change in thermoregulatory efficiency after repeated exposure to heat, can lead to lessened discomfort at the end of summer compared to the start with exposure to the same ambient temperatures (Schweiker *et al* 2018, Meidenbauer *et al* 2024). This ability to evaluate contextual factors and other key processes is vital to better understand how temperature impacts cognition in daily life, and how this may lead to negative clinical outcomes.

This pilot study examines whether fluctuations in thermal comfort influence both subjective and objective cognitive performance in daily life. We examine this using a smartwatch-based *N*-back task in a sample of young adults completing a 2 week EMA study during the summer months. We hypothesized that deviations from preferred temperatures and the resulting discomfort would act as a cognitive load, impairing performance on the *N*-back task. We also hypothesized that similar effects would be seen for self-reported alertness, our measure of ‘subjective’ cognitive functioning. Lastly, we hypothesized that these effects would hold when accounting for key covariates and moderators, specifically, time of day and acclimatization. This pilot study was conducted to both collect new, original data on the effects of environmental distractors using smartwatch-based EMA and ensure that the protocols were sufficiently robust for use in future studies with older adults. Thus, in addition to testing these specific relationships, the current study tests the feasibility of a smartwatch-based EMA protocol to measure fluctuations in cognition related to environmental factors in everyday life.

## 2. Methods

Ethics statement

This study was performed in accordance with the Declaration of Helsinki. This human subjects research study was approved by the Washington State University Institutional Review Board. All participants provided written informed consent to participate in this study.

Participants

Eighteen undergraduate and graduate students (11 male, 7 female) between the ages of 18 and 30 were recruited to participate in this study. As this was a pilot study, we used a convenience sample of individuals who were open to both completing the study procedures and providing feedback on any issues or points of confusion with the protocol.

EMA survey questions

A total of up to 13 questions comprised each EMA survey set. The full list of questions is available in the supplementary materials table S1. For the purpose of this study, five self-report questions were analyzed in addition to the *N*-back task. The questions of interest included: (1) ‘Right now I feel mentally sharp and alert’ (response on a 7-point Likert scale from 1—Not at all to 7—Extremely); (2a) ‘How comfortable is the temperature’ (response on a 5-point Likert scale from 1—Very uncomfortable to 5—Very comfortable); (2b) ‘How does the temperature feel to you’ (forced choice options: Too Cold, Too Hot); (3a) ‘Right now my environment is influencing my performance’ (response on a 7-point Likert scale from 1—Not at all to 7—Extremely); (3b) ‘Right now the most influential environmental factor is’ (forced choice options: Noise, Lighting, Temperature, People, Animals, Smells, Technology). Question 3 was adapted from Kratz *et al* (2020) as a means of evaluating what participants believed to be the most important factor in their environments which may have influenced their cognition (if any).

The participant answered each question by selecting from a dropdown list of options. To minimize the number of questions required, the survey included a branching logic wherein participants’ responses to the primary question would determine if the secondary question was shown. Specifically, when asked about thermal comfort (Question 2a), if either ‘uncomfortable’ or ‘very uncomfortable’ was selected, participants were then prompted with the forced choice question (Question 2b) asking whether they were too hot or too cold. However, if they selected ‘neutral’, ‘comfortable’, or ‘very comfortable’, the forced choice was not shown. Similarly, if participants selected any response other than ‘1—Not at all’ to the question on external environmental distractors (Question 3a), then its forced choice question (Question 3b) was shown, which asked about which distractors were present.

*N* -back task

The smartwatch-based *N*-back task used in the current study was previously validated by Schmitter-Edgecombe *et al* (2024). The *N*-back stimuli were three shapes: circle, triangle, and diamond, and were presented on the screen one at a time alongside two response buttons: a green ‘yes’ and a red ‘no’. A counter indicated the time left out of 45 s. Each *N*-back trial began by presenting brief instructions: ‘Press YES if the shape is the same as the previous one and NO if it is not.’ Participants pressed the ‘Start’ button to initiate the task, and then one of the three shapes with a 3 s countdown timer appeared, followed by a second shape. On each trial, participants indicated whether the current shape was the same as the prior shape by selecting the ‘yes’ or ‘no’ button as quickly and as accurately as possible. Shapes were presented randomly until the 45 s period ended. Each new shape appeared on the screen approximately 500 ms after the participant responded. The presentation was pseudorandomized such that it did not show the same shape more than twice in a row. Participants were not provided with any feedback about their performance at the end of the task.

Procedure

Upon enrollment, participants were provided with an Apple Series 7 watch which prompted them with a chime sound 4x per day for 14 d to complete the EMA surveys and *N*-back task. Participants were taken through the full set of prompts with a researcher, and given an opportunity to ask any questions before they began the EMA period. Each survey set was sent at a random time during the following four windows: 9:30–11:30, 12:30–14:30, 15:30–17:30, 18:30–20:30. The prompt appeared as text on the smartwatch screen. The watch also vibrated when the prompt was delivered. If the participant did not respond, the prompt repeated every 5 min up to 5 times. The 14 d windows occurred between May and October 2023. Most participants completed the procedures in Pullman, Washington.

Analytic approach

Our main dependent variable of interest is *N*-back score, that is, the number correct during the 45 s block. We also looked at the number of trials attempted which may provide additional insights regarding speed of responses without focusing on accuracy. However, as participants were generally very accurate, there was a very high correlation between score and trials attempted (*r*= 0.95), and it may therefore be hard to differentiate between these two measures.

The primary analytic approach is a set of linear mixed-effects regressions which include participant as a random intercept to account for repeated measures. Our preliminary analyses examine whether there are significant associations between rated alertness and performance on the *N*-back task to examine overlap between our ‘subjective’ and ‘objective’ measures of cognition. Our confirmatory analyses focus on whether temperature experiences predict cognition. Thus, we looked at *N*-back score, *N*-back trials attempted, and self-reported alertness as our outcome variables. Thermal comfort (on a 1–5 scale) was our primary predictor variable.

As both thermal comfort and cognition may fluctuate over the course of the day, we ran additional models predicting each of our outcome variables (*N*-back score, *N*-back trials attempted, alertness) and thermal comfort by time of day, both modeled as hour of day (i.e. 9 −20) and as ordinal category (morning 09:30–11:59, afternoon 12:00–16:49, or evening 17:00–20:30). Subsequently, models were run which incorporated both thermal comfort and our time of day measures to test whether the effects held or were altered when controlling for time of day. Lastly, models were run with the interaction term of time of day and comfort to test whether the effects of time of day moderated any associations with comfort. Notably, for ordinal variables with 3 levels, the models generate estimates and interaction terms for both linear and quadratic effects of time of day.

Similarly, as acclimatization can impact thermal comfort, particularly for hot weather over the summer period, we estimated the effects of acclimatization using the month that participants started their participation (i.e. May–October). We first conducted a regression predicting comfort by month, and subsequently, month was included in the regression models predicting *N*-back score, *N*-back trials attempted, and alertness by thermal comfort to see whether the effects held when accounting for potential acclimatization effects. As we expected only linear effects for acclimatization, summer month was coded as a numeric variable rather than treating it as both numeric and ordinal (as in the analyses with time of day). Specifically, we recoded each month as a number from 1–6, where May = 1, June = 2, July = 3, September = 5, and October = 6. No participants were run in August. A regression was first run to test whether comfort was affected by month. Next, mixed-effects models were run predicting the outcome variables from both thermal comfort and month, and lastly, we ran a set of models including their interaction.

We also examined whether distracting temperature led to worse *N*-back performance and alertness ratings. In this model, the lack of any selected distractor was treated as the reference level, and each of these distractors was a categorical predictor. The distractors included temperature, alongside animals, lighting, noise, people, smells, and technology.

Descriptive statistics for all variables of interest can be found in the supplementary materials table S2. All analyses were conducted using R Studio (R Core Team 2018). The packages ‘lme4’ (Bates *et al* 2015) and ‘lmerTest’ (Kuznetsova *et al* 2017) were used to conduct regression models, the ‘psych’ package (Revelle 2024) was used to generate descriptive statistics, and ‘ggplot2’ (Kassambara 2023) was used to generate figures. Regression tables were created using the ‘jtools’ package (Long 2022). Post-hoc power analysis was conducted using the ‘simr’ R package (Green and MacLeod 2016), which calculates observed power for mixed-effect models. The data, R analysis code, and Rmarkdown output can be accessed on the project’s OSF repository: https://osf.io/2wkpc/

## 3. Results

Data characteristics and compliance

Of the 1008 survey sets sent during the study period, participants responded to 797 survey sets (79% compliance), which were used in subsequent analyses. Due to the branching logic of some questions, not all participants completed every possible question. If participants selected either ‘uncomfortable’ or ‘very uncomfortable’ for the thermal comfort question, participants were prompted to indicate whether they were too hot or too cold. Upon preliminary examination of the data, it was found that the secondary follow-up question to thermal comfort only had 60 responses, 45 out of 60 indicated temperatures that were too hot and 15 out of 60 that were too cold. Thus, we were underpowered to separately examine the effects of uncomfortable cold separately from those of uncomfortable heat, and instead, we focus on thermal discomfort overall. For our analysis examining the effects of environmental distractors, it is worth noting that some distractors were more frequently noted than others. Of the 797 responses collected, 248 did not indicate the presence of a distracting environmental factor, 17 indicated the presence of animals, 12 indicated lighting, 159 indicated noise, 228 indicated people, 6 indicated smells, 97 indicated technology, and 30 indicated temperature. Thus, we likely have better statistical power to detect the effects of noise or people, and comparatively low power to detect the effects of animals, lighting, or smells.

Lastly, while it would have been ideal to link weather conditions to the surveys to generate estimates of objective ambient temperature to supplement the thermal comfort evaluations, the majority of surveys were completed indoors and participants were not equipped with any kind of temperature sensor, minimizing this possibility: 50 surveys were completed inside a community building, 12 at a restaurant, 361 at home, 61 in transit, 28 at a park or in nature, 19 at a store, 230 at work, and 36 in another location not listed.

Subjective & objective cognition

As expected, the regression model predicting *N*-back score from reported alertness was positive and significant (*p*< 0.001). However, alertness did not predict more *N*-back trials attempted (*p* = 0.23), underscoring that mental clarity impacts performance quality rather than task volume. Full model results can be seen in the supplementary materials table S3.

Thermal comfort

**Figure 1.**
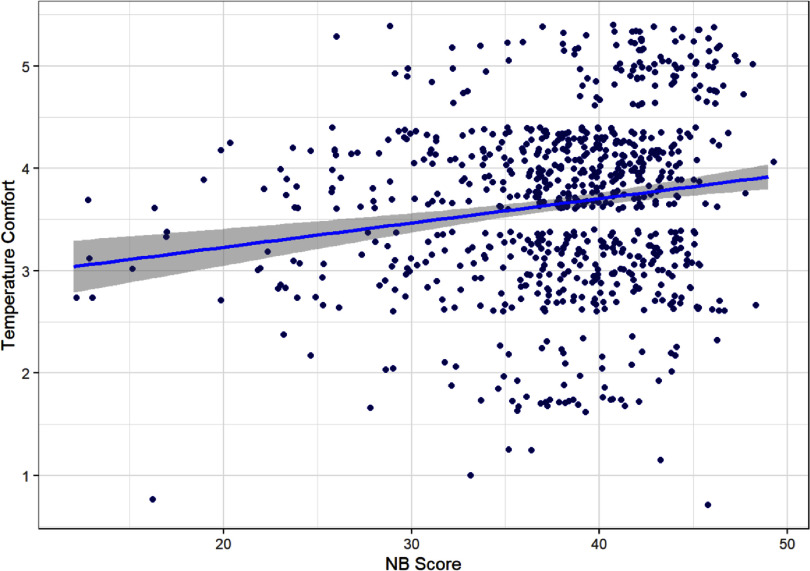
Scatterplot of thermal comfort by *N*-back score across all EMA surveys.

Results of the regression model predicting *N*-back score from thermal comfort showed a positive association between thermal comfort and better performance (*B* = 0.55, *p*= 0.027). The scatterplot in figure 1 shows the relationship between these variables across participants, not accounting for repeated measures. Thermal comfort also predicted more *N*-back trials attempted (*B* = 0.52, *p* = 0.039). In contrast, thermal comfort did not significantly predict higher levels of mental alertness (*p* = 0.072). Full model output for the three analyses can be seen in table 1.

**Table 1.** Linear mixed-effects models predicting cognition by thermal comfort. Regression table reflects unstandardized estimates (B) with 95% CIs.

	*N*-back score	*N*-back attempted	Alertness
Intercept	35.43 ***	36.83 ***	4.01 ***
	[32.73, 38.14]	[34.57, 39.10]	[3.34, 4.69]
Temperature comfort	0.55 *	0.52 *	0.10
	[0.06, 1.04]	[0.03, 1.01]	[−0.01, 0.21]

*N*	797	797	791
*N* (Participant)	18	18	18
AIC	4840.8	4846.6	2436.1
BIC	4859.6	4865.3	2454.8
*R*^2^ (fixed)	0.005	0.006	0.003
Pseudo-*R*^2^ (total)	0.455	0.275	0.544

*** *p* < 0.001; ** *p* < 0.01; * *p* < 0.05.

Time of day effects

Time of day was modeled both as nearest hour (9–20) and as an ordinal factor (morning, afternoon, evening), and regressions were conducted to examine the relationship between outcome variables and time of day, between thermal comfort and time of day, and test for any interaction between comfort and time of day in predicting cognition. There were no significant relationships between score and hour of day (*p* = 0.57) or time of day (linear effect *p* = 0.90, quadratic effect *p*= 0.42). *N*-back trials attempted showed a similar set of null effects for hour (*p* = 0.86) and time of day (linear *p*= 0.86, quadratic *p* = 0.40). However, alertness showed a significant negative relationship with hour (*B* = −0.07, *p* < 0.001) and linear time of day (*B* = −0.45, *p*< 0.001) but not quadratic time of day (*p* = 0.23), suggesting an overall decrease in alertness over the course of the day. See table S4 in supplementary materials for full model output. For thermal comfort, there was not a significant relationship with hour (*p* = 0.61) or a linear relationship with time of day (*p* = 0.44), however, there was a significant quadratic association between comfort and time of day (*B* = 0.09, *p*= 0.03), wherein comfort initially decreased in the afternoon then increased during the evening. Full model results are shown in the supplementary materials table S5.

Next, the time of day measures were included in the main regression models to determine whether the effects held when accounting for these fluctuations in alertness and comfort over the course of the day. Neither hour nor time of day affected the results predicting *N*-back score, *N*-back trials attempted, or alertness from thermal comfort. Lastly, when an interaction term was included between thermal comfort and time of day, the models yielded no significant interactions. Full results for the models with the interaction term are shown in tables 2 and 3. Results from the simple effects models (without interaction) are in the supplementary materials in tables S6 and S7. Thus, while comfort and alertness were each affected by time of day, accounting for these time of day effects did not impact the overall relationships observed.

**Table 2.** Linear mixed-effects models predicting cognition by hour and thermal comfort. Regression table reflects unstandardized estimates (*B*) with 95% CIs.

	*N*-back score	*N*-back attempted	Alertness
Intercept	33.20 ***	33.44 ***	5.78 ***
	[25.27, 41.13]	[25.58, 41.30]	[4.02, 7.53]
Temperature comfort	1.27	1.47	−0.10
	[−0.75, 3.29]	[−0.57, 3.51]	[−0.54, 0.34]
Hour	0.15	0.22	−0.12 *
	[−0.34, 0.64]	[−0.27, 0.72]	[−0.22, −0.01]
Temp comfort x hour	−0.05	−0.06	0.01
	[−0.18, 0.08]	[−0.19, 0.07]	[−0.02, 0.04]

*N*	797	797	791
*N* (Participant)	18	18	18
AIC	4851.67	4857.29	2420.40
BIC	4879.75	4885.38	2448.44
*R*^2^ (fixed)	0.01	0.01	0.02
Pseudo-*R*^2^ (total)	0.46	0.28	0.56

*** *p* < 0.001; ** *p* < 0.01; * *p* < 0.05.

**Table 3.** Linear mixed-effects models predicting cognition by time of day and comfort. Regression table reflects unstandardized estimates (*B*) with 95% CIs.

	*N*-back score	*N*-back attempted	Alertness
Intercept	35.27 ***	36.72 ***	4.23 ***
	[32.44, 38.11]	[34.30, 39.13]	[3.54, 4.92]
Temperature comfort	0.60 *	0.55 *	0.06
	[0.07, 1.13]	[0.02, 1.09]	[−0.06, 0.18]
Time of day (Linear)	1.48	1.81	−0.89 *
	[−1.85, 4.81]	[−1.55, 5.18]	[−1.61, −0.17]
Time of day (Quadratic)	0.02	0.44	0.38
	[−2.58, 2.63]	[−2.20, 3.07]	[−0.19, 0.95]
Temp comfort x time (Linear)	−0.40	−0.47	0.12
	[−1.28, 0.47]	[−1.35, 0.42]	[−0.07, 0.31]
Temp comfort x time (Quad)	0.04	−0.07	−0.08
	[−0.65, 0.74]	[−0.77, 0.64]	[−0.24, 0.07]

*N*	797	797	791
*N* (Participant)	18	18	18
AIC	4848.64	4853.66	2422.76
BIC	4886.09	4891.11	2460.14
*R*^2^ (fixed)	0.01	0.01	0.02
Pseudo-*R*^2^ (total)	0.46	0.28	0.56

*** *p* < 0.001; ** *p* < 0.01; * *p* < 0.05.

Acclimatization effects

**Figure 2.**
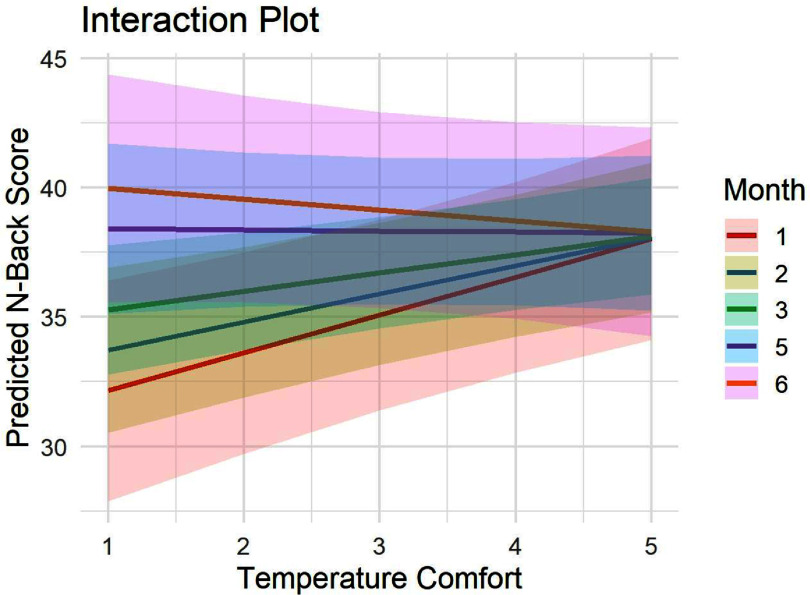
Prediction plot for the model estimating *N*-back score from thermal comfort & month.

Surprisingly, regressions predicting thermal comfort by summer month did not show a significant relationship (*p* = 0.73), however, this may be due to a subset of the thermal discomfort ratings reflecting being too cold (15 cold vs 45 hot), and due to the limited ability to detect a primarily between-participant effect. However, the models predicting *N*-back score and trials attempted by the interaction of thermal comfort and month both yielded significant interactions (*N*-back score *B* = −0.38, *p*= 0.02; *N*-back trials attempted *B* = −0.33, *p* = 0.036) (table 4). Results from the simple effects models (without interaction) are in the supplementary materials table S8. As can be seen by the prediction plot reflecting the interaction in figure 2, the relationship between thermal discomfort and *N*-back score was stronger in the first 3 months (May–July) than the last two (September–October). The interaction was not significant for alertness, however. This does suggest that acclimatization may play a role in reducing the effects of thermal discomfort-related reductions in objective cognitive functioning.

**Table 4.** Linear mixed-effects models predicting cognition by month and comfort. Regression table reflects unstandardized estimates (*B*) with 95% CIs.

	*N*-back score	*N*-back attempted	Alertness
Intercept	28.74 ***	32.03 ***	3.07 ***
	[22.55, 34.94]	[26.75, 37.31]	[1.53, 4.61]
Temperature comfort	1.84 **	1.64 **	0.20
	[0.69, 2.99]	[0.49, 2.80]	[−0.06, 0.46]
Month	1.94 *	1.40 *	0.27
	[0.33, 3.56]	[0.02, 2.78]	[−0.13, 0.67]
Temp comfort x month	−0.38 *	−0.33 *	−0.03
	[−0.68, −0.07]	[−0.64, −0.02]	[−0.10, 0.04]

*N*	797	797	791
*N* (Participant)	18	18	18
AIC	4838.95	4847.56	2445.04
BIC	4867.04	4875.65	2473.08
*R*^2^ (fixed)	0.03	0.01	0.03
Pseudo-*R*^2^ (total)	0.47	0.30	0.56

*** *p* < 0.001; ** *p* < 0.01; * *p* < 0.05.

Distracting temperatures and other environmental factors

The next set of regression models predicted each outcome by the presence of any of one of seven external environmental distractors: animals, light, noise, people, smells, technology, and temperature. Model output for all distractors can be seen in table 5. Of key relevance to the current work, distracting temperature was a significant negative predictor of *N*-back score (*p* = 0.015), but not *N*-back trials attempted (*p*= 0.055) nor alertness (*p* = 0.059). However, it should be noted that these null results may be an issue of statistical power, given the low frequency of temperature as a selected distractor (*n* = 30). Additionally, though the focus of this analysis is thermal distraction, it is worth noting that most of the environmental distractors tested had a negative impact on one or more aspects of cognition.

**Table 5.** Linear mixed-effects models predicting cognition by environmental distractors. Regression table reflects unstandardized estimates (*B*) with 95% CIs.

	*N*-back score	*N*-back attempted	Alertness
Intercept	38.93 ***	40.02 ***	4.65 ***
	[36.77, 41.09]	[38.50, 41.54]	[4.10, 5.20]
Animals	−4.12 **	−3.63 **	−0.34
	[−6.56, −1.67]	[−6.11, −1.14]	[−0.89, 0.21]
Light	−1.94	−1.02	−0.58
	[−4.78, 0.90]	[−3.90, 1.87]	[−1.22, 0.06]
Noise	−1.08 *	−0.85	−0.33 **
	[−2.08, −0.08]	[−1.87, 0.17]	[−0.56, −0.11]
People	−3.03 ***	−2.52 ***	−0.40 ***
	[−3.96, −2.11]	[−3.46, −1.58]	[−0.61, −0.19]
Smells	−2.52	−5.37 *	0.08
	[−6.55, 1.52]	[−9.47, −1.28]	[−0.83, 0.99]
Technology	−1.82 **	−1.76 **	−0.53 ***
	[−3.01, −0.62]	[−2.98, −0.55]	[−0.80, −0.26]
Temperature	−2.45 *	−1.97	−0.43
	[−4.43, −0.47]	[−3.97, 0.04]	[−0.88, 0.02]

*N*	797	797	791
*N* (Participant)	18	18	18
AIC	4799.47	4815.08	2434.04
BIC	4846.28	4861.89	2480.77
*R*^2^ (fixed)	0.04	0.04	0.02
Pseudo-*R*^2^ (total)	0.49	0.30	0.55

*** *p* < 0.001; ** *p* < 0.01; * *p* < 0.05.

Post-hoc power analysis

As this was a pilot study, we did not conduct an *a priori* power analysis to determine the required sample size for our primary analyses. However, to evaluate our power to detect each of the effects of interest in the study, we ran a post-hoc power analysis for multi-level models. We ran 1000 simulations to generate mean observed power values and 95% CIs for each model. The observed power for the models with thermal comfort as a predictor showed 60.8% (95% CI: 57.7, 63.8) power for *N*-back score, 55.00% (95% CI: 51.86, 58.11) for *N*-back trials attempted, and 41.70% (95% CI: 38.62, 44.83) for alertness. Based on these estimates, our sample (18 participants, 797 observations) is below what is required to achieve 80% power with alpha = 0.05. Our power is better for *N*-back score relative to trials attempted or alertness. In light of the strong association between alertness and *N*-back performance, and the trend *p*-value for the alertness-comfort regression, it is very possible that alertness could be significant with a larger sample, warranting clear caution in interpreting these null results. However, the observed power for models with environmental distractors as a predictor all exceeded the 80% threshold: with 100.0% (95% CI: 99.63, 100.0) for *N*-back score, 99.70% (95% CI: 99.13, 99.94) for *N*-back trials attempted, and 97.20% (95% CI: 95.98, 98.13) for Alertness.

## 4. Discussion

While the effects of extreme temperatures on cognitive functioning are well-established in the experimental literature, the extent to which everyday thermal discomfort impairs cognition remains unclear. Using smartwatch-enabled EMA and the *N*-back task, this pilot study demonstrates the feasibility of examining these relationships in an ecologically valid setting. Further, it provides initial evidence that thermal discomfort in daily life does indeed inhibit objective cognitive performance. Thus, it appears that thermal discomfort in typically experienced conditions is a promising proximal mechanism underlying the correspondence between modest ambient temperature changes and population-level effects in mental health conditions and aggressive behaviors.

The relationship between uncomfortable, distracting temperature and cognition was most consistent for *N*-back score, which reflects both speed and accuracy or performance quality, compared to *N*-back trials attempted, or mental alertness, which is a subjective rating. These results are consistent with prior work by Gaoua *et al* (2012) suggesting that thermal discomfort may work, in part, by distracting individuals from an attention-demanding task. The null results for alertness are quite hard to interpret given the comparatively low power to detect an effect in this sample, however. Feelings of confusion or lethargy have been repeatedly documented under inductions of extreme temperatures (Yaqub and Al Deeb 1998, McGarr *et al* 2023, Podstawski *et al* 2023), and it is possible that the weak, non-significant association here indicates that changes in alertness may be tied to more extreme exposures. As our post-hoc power analyses suggested better statistical power to detect the effects on *N*-back performance rather than alertness across analyses, these results should be given considerably more weight in terms of the observed pattern of results. Importantly, the observed power estimations are directly tied to the effect sizes obtained in the current data, and it is unclear what the effect size would be in a larger sample. Accordingly, future work with an adequately powered sample would be essential to test the specificity of the effects on objective vs. subjective metrics of cognition.

Notably, the core finding of this work links cognition to perceived thermal comfort, rather than temperature itself. Due to the lack of an objective temperature estimate to link with the EMA data in this study, the results do not account for the notable variability in thermal comfort across individuals, even in the same conditions (Nicol 2017, Wang *et al* 2018). Variability in thermal comfort may arise from biological (i.e. body mass, recent exercise) and psychological influences (i.e. perceived control, current mood), leading to variability both between individuals and within individuals in a given situation (Zhou *et al* 2014, Schweiker *et al* 2018, Wang *et al* 2018, Özbey *et al* 2023). If discomfort does indeed play a role, the clear variability in the exposure-comfort relationship suggests it is unlikely to be a straightforward, direct link between temperature and cognition.

These results complement those obtained by Meidenbauer *et al* (2024), demonstrating an association between thermal discomfort and worsened affect, but no association between actual temperature conditions and affective states. Based on those results, Meidenbauer *et al* (2024) proposed that the appraisal of a given temperature as uncomfortable may be necessary for ambient conditions to have an impact on emotional states. Nevertheless, it remains an open question whether ambient temperature changes need to first be perceived as uncomfortable for it to lead to an analogous impairment in cognition.

This would be consistent with the idea put forth by Gaoua *et al* (2012), suggesting that sensory displeasure acts as a cognitive load or distractor. In contrast, at extreme temperatures that generate a robust, metabolically-intensive thermoregulatory response or induce hyperthermia, regional alterations in neural activity can lead to disrupted connectivity in networks needed to engage in demanding cognitive tasks (Qian *et al* 2013, Sun *et al* 2013). However, these neurobiological changes would not be expected with more modest changes in ambient temperature, suggesting distinctive pathways to cognitive impairment at different exposure conditions. It would be important in future studies to examine whether this is indeed the case for everyday heat-related cognitive impairment by combining EMA with remote sensing to generate thermal exposure estimates.

Though time of day did show a quadratic relationship with thermal comfort, the results of the analysis incorporating time of day as a covariate and a moderator did not affect the relationship between discomfort and *N*-back performance. This may be, in part, that we did not see any changes in *N*-back performance as a function of time of day. However, it may also be due to the fact that the shape of the effects on comfort and mental alertness were different. Thermal comfort did appear to change throughout the day following an inverted U-shape (higher in the morning and evening, lower in the afternoon), whereas alertness followed a linear relationship. Thus, these time of day effects may exert some influence on comfort and alertness, but they do not appear to fundamentally affect the thermal comfort-cognition link in these data. However, to more thoroughly test this idea, future research with more prompts per day and a larger sample would be highly valuable.

Interestingly, acclimatization did moderate the relationship between thermal comfort and *N*-back performance. Specifically, though thermal comfort itself was not different between months (potentially due to variability in actual exposure that cannot be examined with the current data), the relationship with thermal comfort and *N*-back performance was stronger in early summer months compared to late summer. These results should be interpreted with caution, however, as our measure of acclimatization was a coarse one (summer month of participation), and we did not have many repeated measures for each month. Additionally, there are other differences in season that could be at play here other than acclimatization. Nevertheless, it is interesting to note that a similar analysis examining changes in affect by comfort and acclimatization effect in Meidenbauer *et al* (2024) did not show an analogous effect—while comfort did change over time, it did not moderate the relationship between thermal discomfort and worsened affect. It is possible that the distracting nature of thermal discomfort may attenuate over time as individuals are repeatedly exposed to it, leading to this pattern of effects. However, additional research would be required to explicitly test this potential explanation.

While the study sample of healthy young adults lacks generalizability to the broader population, it is interesting that these results were found in a sample of individuals with low heat or cold vulnerability. Extreme temperature risk is driven, in part, by how effectively an individual can thermoregulate to keep their core body temperature in a healthy range (Saghiv and Sagiv 2020, Pearson *et al* 2023). Age plays a major role, with young children and older adults being of particular high risk, as well as individuals who are pregnant, have pre-existing medical conditions, or are taking medications that reduces thermoregulatory ability (Balmain *et al* 2018, Lõhmus 2018, Millyard *et al* 2020, Pearson *et al* 2023, Taliercio 2024). That we found an effect in a low-risk sample suggests that the effects may be even more pronounced in other, more vulnerable populations, where thermal discomfort is more likely even at more typical conditions.

In addition to the homogenous sample and lack of objective temperature data, our study has a few other notable limitations. First, as this was a pilot study, our sample size is relatively small and some of our null results, particularly those related to self-reported alertness, could certainly be due to insufficient power. These results should be interpreted with caution and highlight the need for an adequately powered study to fully evaluate this relationship. While our study benefits from many repeated measurements per person, future work with larger, more diverse samples is necessary to test the robustness of our effects and generalizability of results. In particular, the effects across different ages, physical and mental health conditions, and climate regions are likely to vary, and should be accounted for in future work.

Second, there are other key variables that may impact the relationship between comfort and cognition, such as emotional states, caffeine consumption, sleep quality, or recent exercise. As part of the EMA procedure, participants were asked about ‘internal factors’ that may have influenced their performance, and one response option was ‘Negative Emotions’. However, in these data, this option was selected very infrequently (21 total occurrences), so examining the effects of negative emotional states was not possible in these data. However, given other research linking thermal discomfort and affect (Gaoua *et al* 2012, Meidenbauer *et al* 2024), and that negative affect can impair cognition (Elliott *et al* 2011), this is certainly a plausible mechanism which could mediate the relationship between thermal comfort and cognitive performance. Investigating this is an important next step, and future studies of this nature would ideally measure affect directly, and incorporate questions about sleep quality, recent exercise, and caffeine consumption to test the importance of these factors.

Finally, as mentioned above, this study speaks to the effects of thermal discomfort, rather than temperature exposure directly. While there is good reason to think that comfort is more important than objective temperature at more modest ranges, the lack of integration with true environmental conditions is a key limitation of this work. In order to fully test the importance of thermal discomfort across the spectrum of temperature ranges, studies which link real-time responses to current thermal exposures are a necessary next step. Given that the average U.S. resident spends over 85% of their time indoors (Klepeis *et al* 2001), and that climate control within indoor areas greatly impacts actual exposure, future studies would ideally directly measure environmental exposures using remote sensing or wearable devices to reduce reliance on meteorological estimates alone.

## 5. Conclusion

In summary, the current work demonstrates that thermal discomfort in daily life can indeed contribute to cognitive decrement in a sample of young adults. This is true even when controlling for the effects of time of day and acclimatization. This work further highlights the feasibility of using the *N*-back task as part of an EMA approach to monitor the associations between environmental factors and fluctuations in cognition. It also opens the doors for future work in different populations, settings, or environmental exposures to understand the cognitive implications of thermal discomfort.

## Data Availability

The data that support the findings of this study are openly available on an Open Science Foundation (OSF) repository at https://osf.io/2wkpc/ (Kimberly 2025). Supplementary Tables available at https://doi.org/10.1088/2515-7620/ae6239/data1.
